# Residents’ Experiences of Learning, Working, and Developing in Entrustable Professional Activity-Based Training

**DOI:** 10.5334/pme.1464

**Published:** 2025-05-08

**Authors:** Maaike P. Smit, Janiëlle A. E. M. van der Velden, Reinoud J. B. J. Gemke, Karsten A. van Loon, Matthijs de Hoog

**Affiliations:** 1 Erasmus MC-Sophia Children’s Hospital, Department of Neonatal & Pediatric Intensive Care, Division of Pediatric Intensive Care, University Medical Center Rotterdam, The Netherlands; 2 Radboud University Medical Center Amalia Children’s Hospital, Nijmegen, The Netherlands; 3 Amsterdam University Medical Center, The Netherlands; 4 Department for Postgraduate Medical Training, Amsterdam University Medical Center, The Netherlands

## Abstract

**Introduction:**

Entrustable Professional Activity (EPA)-based residency programmes are designed to offer structure, flexibility, and a gradual increase in autonomy. While residents are expected to take an active role in their learning, little is known about how they actually experience learning and working within the EPA framework. This study explores paediatric residents’ experiences of learning, working, and developing within an EPA-based training programme.

**Methods:**

We conducted a qualitative study using semi-structured interviews with 11 paediatric residents from three of the seven Dutch training regions. Interviews were transcribed verbatim, anonymised, and analysed using template analysis to identify themes related to residents’ learning and professional development within EPA-based training.

**Results:**

Residents described increasing confidence and competence in the phase prior to entrustment. Some linked this development to the EPA structure, as it supported goal setting and feedback-seeking, while others attributed this development primarily to learning through clinical experience.

The entrustment decision process—particularly the Clinical Competence Committee (CCC) feedback—was seen as reassuring, though residents also described discomfort with being evaluated by a group. After entrustment, residents experienced greater autonomy but noted variation in supervision practices. Some felt unsure about when to request supervision, particularly in apparently straightforward settings. Others described feeling empowered to pursue individualised learning opportunities.

**Discussion:**

In reflecting on these findings, we drew on the concept of Self-Regulated Learning to explore how residents engaged with their training. Making these principles more explicit within EPA-based programmes may support residents in optimising their learning and strengthen their preparation for independent practice.

## Introduction

As competency-based medical education continues to develop, the way residents experience learning within structured training frameworks remains an important area of exploration.

Entrustable Professional Activities (EPAs) form the cornerstone of competency-based residency training. EPAs are units of professional practice, defined as key tasks that residents must progressively learn to perform independently. EPAs are entrusted once residents have demonstrated sufficient competence within defined areas of clinical practice [1,2]. This approach aims to facilitate a gradual increase in responsibility and autonomy during training, while maintaining tailored supervision and high standards of patient care [3]. Many residency programmes worldwide have adopted EPA-based structures, contributing to a growing understanding of how professionals experience learning and working within these frameworks [2,3,4,5,6]. Typically, residents begin with close supervision and gradually have more autonomy to perform professional tasks independently, with supervision remaining available upon request [1]. Moreover, EPA-based programmes facilitate individualised learning pathways, allowing residents to progress at a flexible and personalised pace [7]. The structured design requires residents to take an active role in their learning in order to navigate their training successfully [4].

Ideally, a residency training programme fosters active learner engagement, encouraging residents to take responsibility for their learning and professional development [8]. Although EPA-based frameworks provide structure and flexibility, little is known about how residents experience learning within these programmes. In particular, there is limited insight into how such structures support or hinder residents in developing autonomy, engaging with feedback, and managing their own progression. This qualitative study explores paediatric residents’ experiences of learning, working, and development in the clinical workplace within an EPA-based training programme.

## Method

### Setting

To achieve the aim of this study, we focused on the first national EPA-based residency training programme in the Netherlands, known as *Training Optimisation for Postgraduate Paediatrics* (TOP). In 2017, the TOP programme was implemented across all seven residency training regions in the Netherlands [9]. Each region includes several general hospitals and a university medical centre (UMC). At any given time, approximately 300 paediatric residents are enrolled in this workplace-based training programme, representing the entire national population of paediatric residents.

### EPA-based Residency Training Programme

The TOP programme includes nine generic EPAs, developed according to national and international guidelines [1,10]. At the start of their training, residents are introduced to the EPA framework, including its structure, learning goals, and expectations. Throughout the programme, they are supported by structured supervision, workplace-based feedback, and regular assessment activities to guide their professional development. During their rotations in both general and academic hospitals, residents are progressively exposed to the full range of EPAs. The required level of supervision depends on the resident’s level of competence and stage of training [11]. Entrustment decisions follow a national, standardised procedure. Residents collect evidence of competence in a digital portfolio aligned with the national training plan, including supervisor feedback, test results, and progress evaluations. Once sufficient evidence has been gathered, a Clinical Competence Committee (CCC) meeting is scheduled, in which a group of trained faculty members discusses the resident’s progress and functioning [1]. The meeting concludes with a formal entrustment decision: if the EPA is entrusted, the resident is considered competent to perform the activity independently, while supervision remains available upon request [11,12]. The EPA-based structure of the TOP programme is designed to facilitate personalised learning trajectories. This allows residents to further strengthen their skills with greater autonomy, and to engage in extracurricular or personal learning activities that support their professional development [1,4].

### Study design and participants

This qualitative study was conducted from a constructivist perspective, aiming to explore paediatric residents’ experiences of learning and working within the EPA-based residency training programme in the Netherlands [13]. Semi-structured in-depth interviews were used to gather detailed, individual narratives. The study took place across three national teaching regions—Amsterdam (northwest), Rotterdam (southwest), and Nijmegen (mid-east)—selected to represent different types of hospitals and geographic areas within the country. We aimed for an even distribution of participants across these regions. All eligible residents received an invitation email from an independent administrative assistant containing information about the study. Residents could contact MS or KvL with questions and register via email if they were interested in participating. After a reflection period, written informed consent was obtained. Participation was voluntary, with no incentives offered, and residents were free to withdraw at any time. To reduce the chance of socially desirable responses or unintentional pressure to take part, only KvL—who is not involved in paediatric training—had access to the participant list. Residents were eligible if they had completed at least one full EPA cycle, including evaluation by the CCC. Thirteen residents initially registered; two withdrew before being interviewed, resulting in eleven participants. KvL conducted all interviews online between April and August 2023.

The interview guide was designed to align with the structure of the EPA-based training programme, focusing on three key phases: the period before entrustment, the entrustment decision-making process, and the phase following the entrustment of an EPA. Questions were open-ended and explored learning experiences, supervision, and feedback. After the first eight interviews, minor adjustments were made to the order of questions to encourage more detailed responses in line with the initial intention and focus of the guide.

### Analysis

The audio-recorded interviews, each lasting approximately 45 minutes, were transcribed verbatim by a professional transcription company to ensure accuracy. To maintain participant confidentiality, MS anonymised the transcripts by removing personal identifiers such as supervisor names and workplaces. Qualitative data analysis software (Atlas ti^®^) was used to facilitate the analysis. We used template analysis, a structured yet flexible form of thematic analysis described by King et al. that allows for hierarchical coding while remaining adaptable to the study’s objectives [14,15]. KvL and MS independently coded a subset of the transcripts, discussing discrepancies iteratively to refine the coding scheme. Following discussions within the research team, a final coding template was established and subsequently applied to all data by MS.

### Social constructivist approach

Social constructivist theory recognises the coexistence of multiple realities and describes how individuals construct new meaning based on their past experiences. This perspective is particularly well-suited to explore learning in complex healthcare environments, as it allows an in-depth understanding of how residents’ personal experiences and perceptions shape their learning [13].

### Reflexivity in the research team

The research team consisted of five researchers with diverse backgrounds and expertise in qualitative research and medical education. The first author (MS) is both a PhD student and paediatric resident. KvL is a senior educational scientist, not involved in paediatric training. MdH, RG, and JvdV are paediatricians and (former) programme directors, each involved in the national development, implementation, and evaluation of the TOP programme, endorsed by the Dutch Association of Paediatrics.

Throughout the study, the team practised reflexivity by regularly discussing their perspectives, analytical decisions, and emerging interpretations [16,17]. The researchers’ prior experiences informed various stages of the study, including the development of the interview guide and the interpretation of findings. These perspectives were made explicit and critically examined during collaborative analysis and team discussions [17].

To broaden the interpretative lens and reduce potential bias, preliminary findings were also discussed in an online panel meeting with three independent experts: an educational scientist, a psychologist, and a resident. Their reflections informed our interpretation of the results and helped to enrich the discussion.

## Results

The EPA-based training structure informed both the interview design and the organisation of the results. Presenting the findings along key training phases offers a structured view of how residents experience their learning, working, and development throughout their training.

### Training phase before the entrustment of an EPA

Residents described growing confidence, self-reliance, and competence during the training period prior to the scheduled CCC meeting. Some felt that the EPA structure supported this development by encouraging them to set concrete learning goals and actively seek feedback (Quotations 1 and 2, Table 1). Others, however, perceived that these skills emerged more organically through clinical experience, rather than being directly facilitated by the EPA framework. As residents gained more clinical exposure, most reported a gradual increase in autonomy and a declining need for supervision. This change felt like a natural progression and was not always explicitly linked to the EPA-based structure. To prepare for the CCC meeting, residents are required to compile a portfolio that aligns with the national training plan. Most did not experience this preparation as difficult or stressful. However, not all residents viewed it as a meaningful learning activity—some described it as a procedural task with limited educational value (Quotation 3, Table 1).

**Table 1:** Training phase before the acquirement of an EPA.


**Q1. (Interview 7, Atlasti 7:10)** It’s a reminder that you need to ask for feedback (…) Maybe today is a good day to ask for feedback? Then you are much more conscious of making a plan. Otherwise, you think, ‘I will ask another day.’ Then months go by without you working on it. **Q2. (Interview 11, Atlasti 15:9 and 15:15)** Now I realise that I can provide input. I can clearly discuss what my learning objectives are (…) For instance, I’m interested in receiving feedback on how I write discharge reports. **Q3. (Interview 11, Atlasti 15:22 and 15:29)** I will only apply for my EPA if I meet that stupid list. (…) I don’t feel any additional pressure or stress to work towards the EPA. It’s a bit of a handle, but not much more than that. It’s really a checklist that I have to check off.


### The entrustment decision-making process of an EPA

After compiling their portfolios and engaging in self-reflection, residents assess their progress towards competence and evaluate whether they feel ready to perform the activity with supervision on request. Following this, residents schedule a meeting with their rotation director. This meeting is almost unanimously described as an informal interim assessment in which residents discuss their readiness to request entrustment for the EPA. When they receive approval to schedule the CCC meeting, this is often perceived as an implicit signal that entrustment is likely, reinforcing their confidence in a positive outcome (Quotation 4, Table 2).

**Table 2:** The entrustment decision-making process of an EPA.


**Q4. (Interview 3, Atlasti 3:11)** I have had a number of assessment meetings (…) I see it as an indicator of my competence for the EPA (…) EPAs are simply guidelines, if you are a good resident, it doesn’t have to be a big deal (…) you will have a CCC meeting and achieve an EPA. **Q5. (Interview 6, Atlasti 6:44)** The most valuable aspect for me wasn’t just the EPA achievement, it was the opportunity to assess everything, a reason to list everything that was going well and what could be improved. **Q6. (Interview 10, Atlasti 14:9 and 14:27)** I want to perform well for the supervisors, especially if I know that they have high standards, I’m more likely to try to meet them (…) It is always a bit nerve-wracking, but I’m curious to hear their feedback and what supervisors will say about me. **Q7. (Interview 2, Atlasti 2:20)** I feel supported knowing that multiple supervisors have discussed this amongst themselves and have entrusted me. I think I appreciate the feeling of achieving an EPA. **Q8. (Interview 4, Atlasti 4:22)** I have never experienced that. I would find it difficult if the EPA was rejected because you can only apply if you really think you can do it. So I would question the preliminary phase, what went wrong? Also, I was not fully aware of how I was functioning. **Q9. (Interview 1, Atlasti 1:19)** I had to return to the department, which I was dreading because I knew it had not gone well the first time. I was afraid of being back in an environment where it did not work last time. Will I succeed this time? I have two months to show it, it has to work out, otherwise, I’m really in trouble. But of course, that is nonsense! I have a lot more experience now, I am older. Everything is going well.


During the CCC meeting, supervisors evaluate the resident’s performance and provide feedback. Many residents highly value this feedback, describing it as meaningful and helpful for both personal and professional reflection (Quotation 5, Table 2). However, some residents find the experience of having their performance discussed by a group of supervisors uncomfortable (Quotation 6, Table 2).

After being entrusted with an EPA, residents commonly described feelings of happiness, confidence, and motivation, marking a significant step toward their goal of becoming a medical specialist (Quotation 7, Table 2). A negative entrustment decision following a CCC meeting was considered rare, with only a few known cases. Nonetheless, even the idea of such an outcome evoked a strong sense of failure. Several residents, despite not having experienced this themselves, speculated that it would likely lead to frustration or disappointment with their supervisors, as they believed that daily feedback should have already clarified their level of competence (Quotation 8, Table 2). One resident who had experienced a negative entrustment decision described similar emotions, including anxiety and pressure to improve performance (Quotation 9, Table 2). However, in hindsight, the resident did not view the delay in entrustment as a failure. Instead, it led to greater awareness of personal learning needs, the formulation of individual learning goals, and more active help-seeking during clinical work. The resident described a positive learning curve and increased confidence—one that, in the residents’ view, would not have occurred if the EPA had been entrusted immediately.

### The phase following entrustment of an EPA

Once a positive summative entrustment decision has been made by the CCC, residents are expected to determine when supervision is needed (Quotation 10, Table 3). While they appreciated the gradual increase in independence leading up to this point, some described feeling unsettled by the autonomy that followed (Quotation 11, Table 3). Several residents felt the need to justify their requests for supervision in relatively straightforward clinical situations, as they were now expected to work independently. Most residents considered independent practice more feasible in general hospitals, where patient care is often perceived as less complex. In contrast, working under remote supervision in UMCs was viewed as more challenging due to the high complexity of cases (Quotation 12, Table 3). In such settings, residents valued the opportunity to work closely with supervisors, appreciating the educational benefits and the sense of safety it provided.

**Table 3:** The phase following the acquirement of an EPA.


**Q10. (Interview 8, Atlasti 8:18 and 8:33)** With your EPA, you can say; I have my EPA! So I can have more independence (…) just to feel a bit more independent or to feel more responsible at a certain point. To work more like colleagues with the paediatricians. You never do it one hundred percent alone. **Q11. (Interview 10, Atlasti 14:15)** I actually tend to discuss things more often. It also helps to have some kind of reassurance from your supervisors that they have confidence that I can do it. It is easier embrace that independence if you have a safety net. **Q12. (Interview 2, Atlasti 2:38)** Because the subspecialty knowledge is quite specific, you actually need a supervisor again. So I’m still finding my way. How can I show more independence during these rotations? **Q13. (Interview 3, Atlasti 3:15)** I said to my supervisor: Should I go first? Or should we go there together? But you will be quiet and observe (…) Give me a bit more responsibility because I achieved this EPA! **Q14. (Interview 7, Atlasti 7:27)** Of course, you are responsible for your own training, and regardless of whether you receive an EPA, you should constantly strive for improvement to become a better paediatrician. That is the ideal approach. The resident is in the lead and is responsible for their training.


At the same time, many residents observed that supervision practices did not always align with the level of entrustment or clinical complexity. According to residents, some supervisors seemed hesitant to allow them to determine when supervision was needed. This mismatch led to frustration among residents—some responded by taking more initiative to work independently, while others accepted the situation to avoid conflict (Quotation 13, Table 3).

Opportunities for individualisation—ranging from small adjustments (e.g., personal learning goals) to larger initiatives (e.g., electives or research)—were perceived to depend more on the work setting than on the EPA framework itself. Several residents found it easier to individualise their learning in general hospitals, where they had more direct contact with the programme director. They also noted that meeting training goals was often more straightforward in these settings, which created space for additional learning initiatives. When successful, such efforts contributed to a sense of accomplishment and support, reinforcing residents’ motivation to pursue their professional ambitions.

This motivation also led some residents to reflect more broadly on their development and future. They described how they were beginning to adopt a mindset of lifelong learning. They saw themselves as actively shaping their own careers and considered the EPA-based programme a useful framework for both professional growth and individualisation. Often, they connected their day-to-day learning to the broader goal of becoming a paediatric specialist capable of delivering excellent care (Quotation 14, Table 3).

## Discussion

This study explored how paediatric residents experience their learning, working, and development within an EPA-based training programme. Our findings show that the way residents engaged with goal setting, feedback, and growing autonomy reflects an active and ongoing involvement in their learning process. Reflecting on this, we recognised strong parallels with the model of Self-Regulated Learning (SRL) as described by Zimmerman (2002) [18]. We therefore use this model to structure the discussion and examine how the phases of SRL may help interpret the ways in which residents navigate their development within the EPA framework. SRL encompasses the cognitive, motivational, and emotional dimensions of learning [19]. Zimmerman conceptualised SRL as a cyclical process consisting of three phases [20,21]. These phases describe how learners set goals and plan strategies (forethought), implement and monitor learning strategies (performance), and evaluate their progress to refine future approaches (self-reflection) [18]. Although we structure the discussion using the SRL framework, we do not suggest a direct alignment between its phases and the formal stages of EPA-based training. Rather, elements of all three SRL phases may emerge throughout residents’ development, depending on the situation and context.

Zimmerman’s forethought phase describes how learners set goals and plan their learning strategies, influenced by motivational factors such as self-efficacy and expected outcomes. When reflecting on our findings, we recognised that residents demonstrated elements of this phase in their training. For example, prior to entrustment, residents often articulated personal learning goals and sought feedback to monitor their progress. Their motivation was frequently linked to the perceived outcome of being entrusted with an EPA. Interestingly, some residents felt that, for example, goalsetting was stimulated by the EPA structure, while others described a more organic development driven by clinical experiences. This suggests that while the EPA framework may encourage forethought processes, residents also relied on situational factors and their own reflective considerations to shape their learning. Similar patterns have been noted in earlier work, which highlights how variation in clinical exposure and informal learning moments can significantly contribute to professional development [22,23].

In the performance phase, residents are actively involved in clinical work and monitor their progress by engaging with feedback. In our study, many residents described using strategies such as time management, task prioritisation, timely feedback-seeking, and reflection to stay engaged and motivated in their training. This aligns with prior research, which has shown that such self-regulation strategies are closely linked to the development of clinical competence and professional identity [24,25]. By working towards specific goals within the EPA framework, residents are able to break down complex clinical tasks into smaller, more manageable steps, an approach that supports gradual growth and further strengthens their self-regulation skills [8]. However, our findings also show that residents often view their progression toward competence as a natural result of increasing clinical experience, rather than as something actively structured by the training programme. While this perspective reflects trust in workplace-based learning, it may reduce opportunities for residents to apply intentional learning strategies [8,25]. When feedback is used actively and constructively, it not only enhances learning within the EPA framework but also contributes to a culture of ongoing improvement and adaptability, both of which are essential in contemporary medical practice [26,27].

In the self-reflection phase, residents reflect on their development and readiness for entrustment, often together with a supervisor. The structure of entrustment decision-making may support residents in goal setting, self-assessment, and feedback-seeking, thereby reinforcing their involvement in strategic learning processes [5]. In our study, most residents described feelings of happiness, confidence, and motivation upon being entrusted with an EPA, while the thought of a delayed entrustment decision evoked emotions such as anxiety, sadness, and disappointment. That delayed decisions are rare raises questions about how entrustment is timed and handled. It may suggest that residents only apply when they are highly confident of success, or that evaluations are not always sufficiently critical. These are concerns that have been noted in previous research on medical assessments [28]. It is also plausible that both supervisors and residents experience discomfort around negative outcomes, contributing to this cautious application behaviour. Nevertheless, our findings suggest that experiencing a delay in entrustment can stimulate meaningful self-reflection and intentional learning. The resident who did not receive entrustment on the first attempt described increased awareness of learning needs, more intentional goal setting, and greater supervision-seeking, providing an example of adequate SRL in practice. This aligns with earlier research suggesting that encountering setbacks can promote resilience and adaptive learning behaviours [29,30]. Rather than viewing delayed entrustment solely as a failure, these experiences may offer valuable opportunities for residents to strengthen their self-regulation and professional growth. Normalising the possibility of delayed entrustment within training programmes could help reduce the stigma surrounding these outcomes and foster a developmental mindset among both residents and supervisors. Ultimately, normalising delayed entrustment could support not only lifelong learning but also a more resilient and reflective medical workforce [27].

Building on insights from the self-reflection phase, our findings also suggest that residents’ reflections may influence how they interact with supervisors and navigate varying levels of supervision and support. Residents described experiencing different degrees of support, communication, and collaboration with supervisors after being entrusted with an EPA. Residents reported that support systems in general hospitals tend to be more informal and less structured, fostering greater autonomy but sometimes accompanied by inconsistent feedback. In contrast, UMCs typically provide more formalised support and structured feedback, ensuring clearer expectations and greater consistency. However, residents noted that the larger scale of UMCs can lead to less personal engagement and a greater sense of social distance, which may impact their comfort and learning experience. These differences may influence the development of SRL skills, as each training environment presents distinct opportunities and challenges for fostering various aspects of self-regulation. Recognising these variations underscores potential areas for improving support systems and communication channels within residency training programmes [26]. Enhancing these aspects is particularly important, as previous studies highlight the crucial role of supportive social relationships, especially with supervisors, in maximising residents’ learning potential. Strengthening communication and feedback practices within different hospital environments may improve residents’ ability to engage with their learning and navigate challenges independently [31].

Beyond these findings, fostering a mindset of lifelong learning remains a core goal of residency training. Professionals who actively reflect on their development and take ownership of their learning are better equipped to adapt to evolving medical knowledge and healthcare demands [32,33]. Our findings suggest that residents begin to adopt this mindset to varying degrees during their training, which aligns with literature highlighting its relevance for ongoing professional development [25]. We argue that consistent support for how residents engage with feedback, reflect on their progress, and direct their own learning can lay a strong foundation for lifelong learning.

### Strengths and limitations

The nationwide implementation of the EPA-based residency programme provides a robust foundation for this study, based on a consistent structure used across training regions. The involvement of an independent panel of experts in the consultation process further enhanced the credibility and trustworthiness of our interpretations.

However, as the study focused exclusively on paediatric residents, the findings may not be directly transferable to other medical specialties where training structures, supervisory practices, or the use of EPAs may differ. Additionally, the cultural and systemic characteristics of the Dutch healthcare and educational system may limit the applicability of these findings to other international contexts. As participation was voluntary, the sample may reflect a relatively motivated group of residents, which could have influenced the perspectives shared.

### Future implications

To support individualised learning and lifelong development, we recommend that residency curricula more explicitly address the development of SRL skills within EPA-based training. Exploring ways to explicitly integrate SRL principles may help residents become more aware of their learning process and strengthen their preparation for independent practice in a changing health care landscape.

Further research could examine how SRL-related strategies affect professional growth over time, and how supervisors can effectively support residents in developing these skills within EPA-based training programmes. Such insights may contribute to more responsive training environments and better preparation for the evolving demands of clinical practice.

## Conclusion

This qualitative study provides insights into how paediatric residents experience their own learning, working, and professional development within an EPA-based residency training programme. Residents described how EPA structures both supported and challenged their autonomy, engagement with feedback, and navigation of their learning. In discussing these findings, we drew on the concept of SRL to interpret patterns of learner engagement that appeared across different phases of training. Our interpretation suggests that making such processes more explicit within EPA-based programmes may help residents optimise their learning and better prepare for independent clinical practice. This may be particularly valuable in fostering lifelong learning and supporting sustainable professional development.

## Abbreviations

CCC: Clinical Competency Committee

EPA: Entrustable Professional Activity

SRL: Self-regulated Learning

TOP: Training Optimisation for Postgraduate Paediatrics

UMC: University Medical Center
